# Recolonization by Slugs: Vertical and Horizontal Dispersal by the Field Slug, *Deroceras reticulatum*

**DOI:** 10.3390/insects12060531

**Published:** 2021-06-08

**Authors:** Gordon Port, Alan Craig, Mark Shirley

**Affiliations:** School of Natural and Environmental Sciences, Newcastle University, Newcastle upon Tyne NE1 7RU, UK; craig.alan45@yahoo.com (A.C.); mark.shirley@newcastle.ac.uk (M.S.)

**Keywords:** movement, slug populations, trap, control, activity

## Abstract

**Simple Summary:**

Slugs are persistent pests. Following control treatments, the numbers of slugs can rapidly return to pre-treatment levels and it is often assumed they are migrating in to the site from adjacent areas. By comparing plots with restricted access to those with open access over a 32-month period we were able to compare the importance of migration from adjacent areas with migration from the soil below the plots. For *Deroceras reticulatum*, which is a major pest species, a large proportion of immigration came from soil below the undisturbed grassland plots. The importance of this inactive subpopulation, below the soil surface, needs further study.

**Abstract:**

Following treatment with molluscicides or other controls, slugs can recolonize a site very quickly, but the proportion of the colonizing slugs moving from adjacent areas (horizontal dispersal) and the proportion from within the soil (vertical dispersal) has not previously been established. At a grassland site, barriers were used to exclude and trap slugs in order to estimate horizontal and vertical movement over a period of 32 months. For the first 15 months vertical movement made a significant contribution to the slugs recolonizing a grassland area. The ecological mechanisms occurring and the implications for the control of slugs are discussed.

## 1. Introduction

Slugs are able to recolonize areas where numbers have been reduced by treatment with molluscicides or nematodes. The rate at which the slug population recovers is affected by weather conditions and, in temperate areas, is rapid when the soil is moist and the temperatures remain above 5 °C. When conditions favour slug activity the population can return to levels which will cause significant damage to crops within two weeks of treatment and, after this time, treatment effects on slug numbers are difficult to detect [1].

Slugs may invade the treated area by movement from adjacent sites (horizontal dispersal), or by upward movement of slugs from within the soil (vertical dispersal). For some large species of slug, such as *Arion vulgaris* Moquin-Tandon, horizontal dispersal can significantly increase slug populations in an area [2] and barriers to horizontal movement can help protect crops [3].

In recently cultivated soil, slugs may be buried during cultivation and gradually return to the surface [4], but, even in the absence of cultivation, some slugs may still be found deep in the soil. In preliminary experiments on a grassland site we found evidence that a substantial proportion of the slug population lived deep within the soil. The present investigation set out to evaluate the size of this subpopulation and to estimate its contribution to recolonization following removal of the surface-active population. To achieve this, we used barriers to collect slugs from defined areas and, on some sites, to prevent recolonization by horizontal dispersal.

Slugs are deterred from crossing barriers made of certain metals such as zinc and copper [5]. This fact has been used to create barriers around defined areas from within which slug activity and numbers can be assessed by trapping from beneath a shelter: a so called Defined Area Trap (DAT) [6,7]. We used permanent and temporary DATs to remove the surface-active population at intervals and to compare recolonization by horizontal and vertical dispersal.

Following removal of the surface-active population we hypothesised:

1. Most recolonization would be by horizontal dispersal.
2. Vertical dispersal would make a small contribution to recolonization and over a short period.

## 2. Materials and Methods

### 2.1. Barriers Used for Trapping

We used three types of trap:

1. A “Temporary” square Defined Area Trap (DAT), 40 × 40 cm with sides 25 cm high made of galvanized steel sheet. The trap sides were pushed into the soil to a depth of 5–10 cm (depending on soil density). For a two-week period, slugs were collected in the morning from beneath a hardboard sheet that lay on the soil surface, fitting within the trap. After two weeks of trapping the DAT was removed for two weeks, to allow horizontal and vertical dispersing slugs to recolonize the site. The trapping was then repeated, returning the DAT to exactly the same location.
2. A trap as in (1) was used, but was not removed (“Permanent”) between trapping periods so that only vertically dispersing slugs were able to recolonize the site.
3. A trap as in (2), but the DAT was closed (“Covered”) with a close fitting lid made of fine mesh. The lid allowed air movement, but ensured that no slugs that climbed the wall of the DAT could enter or leave.

### 2.2. Site Description

The traps were used on a mixed grassland site that had not been cultivated for several years at the Biology Field Station, Close House, Northumberland, UK (54°59′15″ N, 001°48′07″ W). At each sample point one trap of each type was set out in a triangular arrangement with the traps 1.5 m apart. There were six replicate sample points set out in two rows of three, the sample points being 6 m apart.

In most months, over a 32 month period (April 2006–October 2008), slugs were removed from all the traps during the two-week trapping period, identified to species and weighed before releasing them at another site. Identifications were based on Cameron et al. (1983) [8].

### 2.3. Statistical Methods

The numbers trapped included many zero counts and the trends and seasonality were investigated with a Seasonal Decomposition of Time Series by Loess (STL) [9] in R (version 3.6.3) [10] using the ‘stats’ package. Analysis of slug numbers used a Chi square test of association. Data on slug weights were analysed with a GLM ANOVA.

## 3. Results

### 3.1. Slug Species

**Figure 1:**
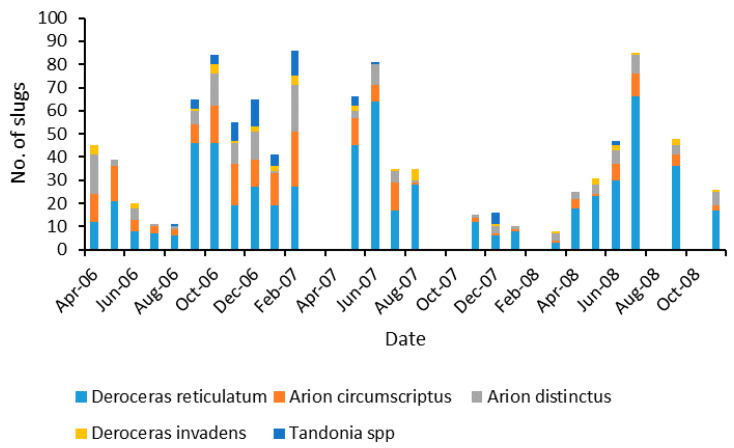
The numbers of different slug species collected from traps at a grassland site over 32 months.

**Figure 2:**
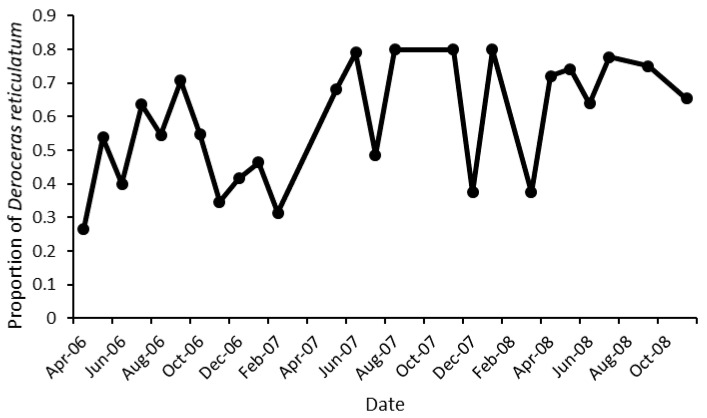
The proportion of *Deroceras reticulatum* in slug species collected from traps at a grassland site over 32 months.

Over 1000 slugs were collected during this experiment with the majority being *Deroceras reticulatum* (Müller) (Table 1). The relative numbers of these species varied throughout the course of the experiment (Figure 1), but *D. reticulatum* usually represented between 30% and 80% of the total numbers collected in any month (Figure 2). The relative numbers of *D. reticulatum* were lower in the winter months.

### 3.2. Trap Captures

As expected, the total numbers of slugs collected from the experiment varied from month to month depending on the weather conditions. Relatively low numbers were collected in the hot, dry summer of 2006, but slugs were abundant throughout the wet summers of 2007 and 2008.

**Figure 3:**
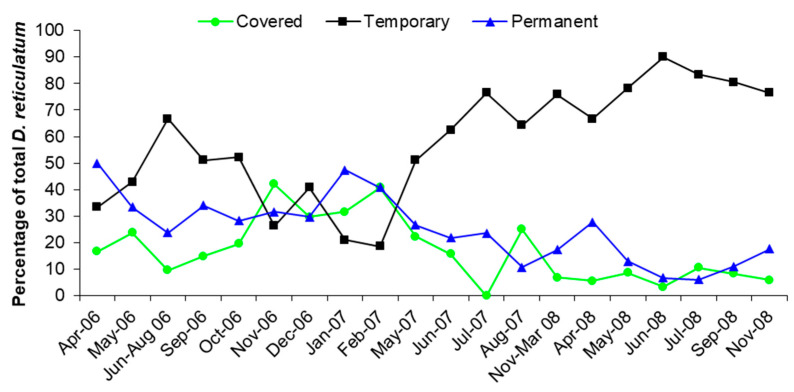
The percentage of the total *Deroceras reticulatum* numbers for each sampling period found in each trap type.

In order to compare the numbers collected from each trap type, whilst accounting for variation in total numbers from month to month, the numbers were recorded as a percentage of the total catch within each month. In order to compensate for periods when low total numbers of slugs were captured, the results for June–August 2006 were combined, as were the results for November 2007–March 2008. The numbers of slugs captured from Permanent and Covered traps were very similar indicating that the number of slugs entering Permanent traps from outside was negligible. Figure 3 shows the percentage of the total *D. reticulatum* numbers found in the traps for each sampling period. The relative percentage of *D. reticulatum* found in the Temporary traps showed a marked increase in the second year of sampling.

**Figure 4:**
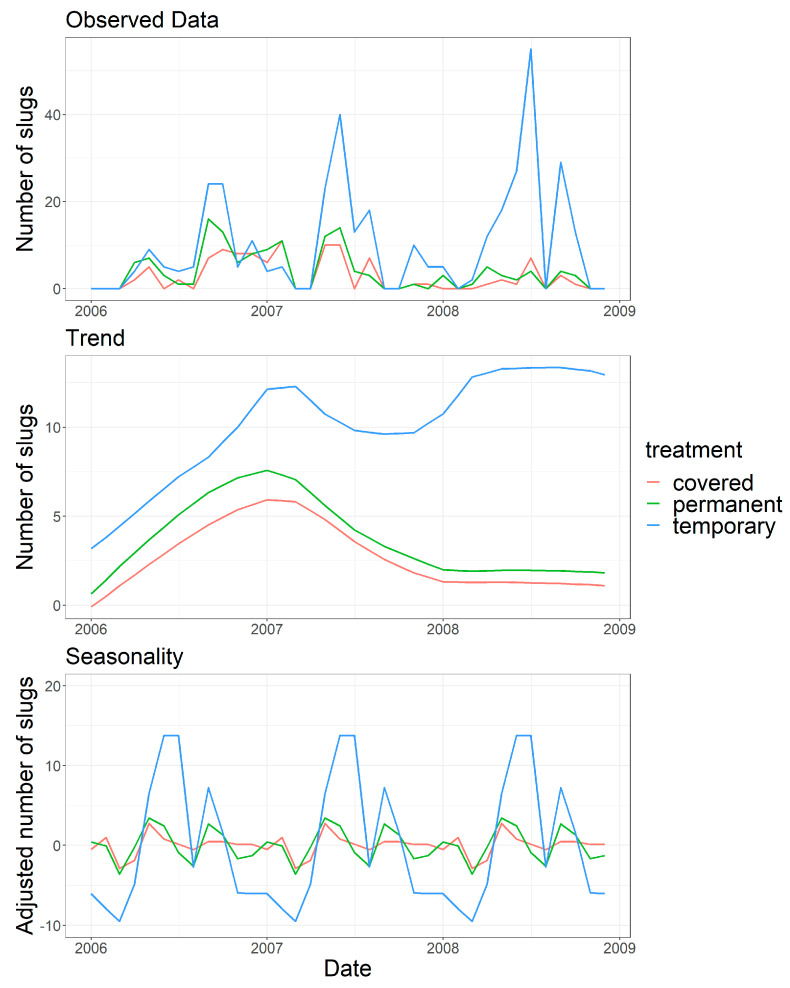
The numbers of *Deroceras reticulatum* collected from traps at a grassland site analysed by Seasonal Decomposition of Time Series by Loess (STL).

Comparing the total numbers of *D. reticulatum* collected in the different trap types in the first 12 months of the study with those collected in months 13–32 showed that the relative contribution of the trap types differed significantly between the two periods (X^2^_(2)_ = 56.874, *p* < 0.001) confirming the trends shown in Figure 3. The STL analysis revealed both the trends in the data and the effect of season (Figure 4). The trends confirm the similar numbers collected in all trap types for the first 12 months of the study. In the following period many more slugs were collected from the Temporary traps.

**Figure 5:**
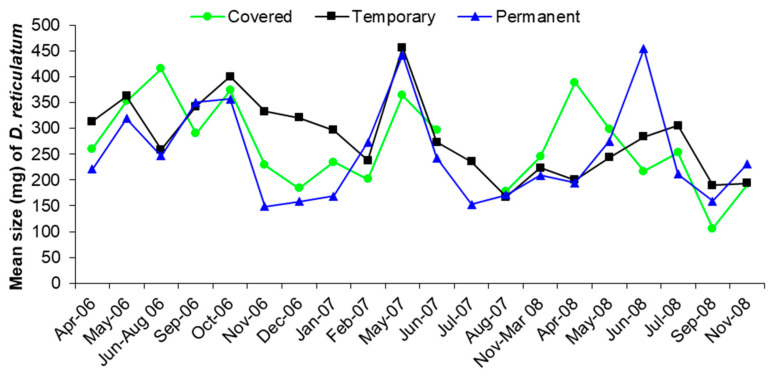
The average size (mg) of *Deroceras reticulatum* for each sampling period found in each trap type.

The average size of *D. reticulatum* in the different trap types was similar (F_2,580_ = 1.46, *p* = 0.232), so there was no evidence that recruitment of small slugs was more important in one trap type or the other (Figure 5).

In each sampling period virtually all the active slugs were removed from within the trap area. We assume that slugs caught in that same area the following month had recolonized (invaded) as a result of dispersal and we can estimate the numbers that dispersed vertically, from within the soil, and those that dispersed horizontally, from adjacent areas.

1. Those slugs dispersing *vertically* were estimated from the average capture in Covered and Permanent traps where horizontal movement was prevented.
2. Those slugs dispersing *horizontally* were estimated from the Temporary traps, where both horizontal and vertical dispersal were possible, after subtracting the number estimated to have dispersed vertically.

**Figure 6:**
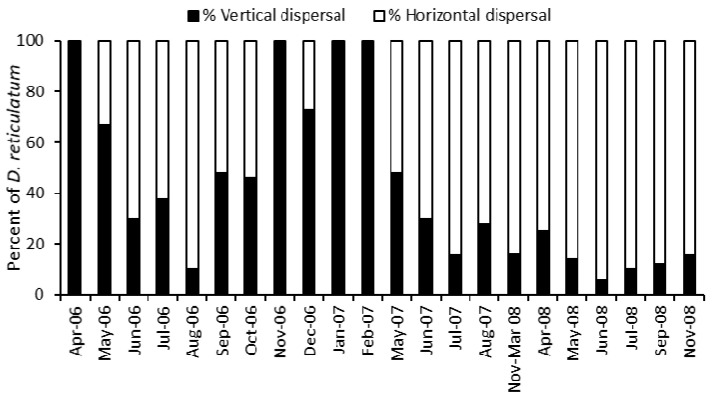
The percentage of *Deroceras reticulatum* invading vertically and horizontally estimated in each sampling period (see text).

Figure 6 shows that, for the first 15 months of trapping and removal, vertical dispersal made an important contribution to the population the following month. The importance of vertical dispersal diminished over the subsequent trapping period.

## 4. Discussion

The results in Figure 3 show that vertical dispersal by slugs within the soil makes a significant contribution to the numbers invading a site after removal of the surface active population. Slugs sampled from the traps were removed from the site and this simulated the effect of active slugs being removed from the population by control measures such as treatments with chemical molluscicides or nematodes. The total numbers of slugs found in the traps showed no consistent decrease with time as slugs recolonized the trap sites by both horizontal and vertical dispersal. The contribution of vertical dispersal, shown by the captures in Covered and Permanent traps, made an important contribution to recolonization for the first year of the experiment and was the major source of new slugs during the winter of 2006–2007 (Figure 5). After May 2007 there was a smaller contribution by vertical dispersal, but this was expected as the slugs had been regularly removed from the traps and there was little opportunity for the population below the surface to be replenished by reproduction or immigration.

As each individual trap in this experiment covered a relatively small area (0.016 m^2^), we expected a large proportion of recolonization to be due to horizontal dispersal. Slugs, such as *D. reticulatum*, move several metres each night in favourable conditions [11], but the rate at which they disperse is probably low [12]. Slugs frequently return to previous daytime resting sites and the rate at which new areas are colonised is not known. The rate of invasion is very likely affected by local environmental conditions, but we consider the mixed grassland, both inside and outside the trapped areas, to provide a favourable habitat and this may have reduced the net horizontal dispersal.

When farmers and growers apply control treatments to land, they often treat large areas and this will minimise the opportunities for recolonization by horizontal dispersal. Our results suggest that recolonization by vertical dispersal was of equal importance to horizontal dispersal in the first 15 months of the experiment, notwithstanding the fact that the monthly collections from the traps represented monthly control treatments in removing the active slugs. When farmers and growers experience control “failures” following a treatment this may be due to the treatment affecting relatively few active slugs, but our results suggest that invasion of the treated area from below ground by vertical dispersal may be a key factor.

We expected the zinc treatment, coating the walls of the DAT, would prevent slugs from entering or leaving the trap area by climbing the walls. To confirm this, we included the third, Covered, trap type in the study. The similar captures made in Permanent and Covered traps show that, even in the absence of a cover the numbers of slugs entering the Permanent traps from outside the area was negligible.

Our results show that for *D. reticulatum*, when surface active slugs are removed from a site by hand collection, new individuals reinvade the site. Furthermore, for the first 15 months of our investigation, a large percentage of the reinvading slugs, in some months 100%, were from the soil below the area. What these slugs are doing below the soil surface is not known. As slugs such as *D. reticulatum* normally move on the soil surface it seems that the subpopulation below the soil surface are not active; whether they are in a quiescent state would be an interesting study, but the difficulties of sampling the subpopulation would make the investigation challenging. That individuals may remain in this quiescent subpopulation for a year or more is also intriguing.

Although our study was limited in its size we were able to continue it for a long period. Our results show that any treatment aimed at killing slugs when applied to the soil surface will only affect a small proportion of those at the site. Following treatments with molluscicides or nematodes the active slug population will be replenished within a few weeks by recruitment from adjacent areas and from a quiescent subpopulation present below the soil.

## Figures and Tables

**Table 1:** Total numbers of different slug species collected from traps at a grassland site over 32 months.

Slug Species	Number
*Deroceras reticulatum* (Müller)	611
*Arion circumscriptus silvaticus* Lohmander	196
*Arion distinctus* Mabille	146
*Tandonia* spp.	57
*Deroceras invadens* Reise, Hutchinson, Schunack &Schlitt	40
*Arion circumscriptus circumscriptus* Johnston	11
*Arion fasciatus* (Nilsson)	1

## Data Availability

The data represented in this study are available on request from the corresponding author.
